# Diffuse smoking-related lung diseases: insights from a radiologic-pathologic correlation

**DOI:** 10.1186/s13244-019-0765-z

**Published:** 2019-07-16

**Authors:** Célia Sousa, Márcio Rodrigues, André Carvalho, Bárbara Viamonte, Rui Cunha, Susana Guimarães, Conceição Souto de Moura, António Morais, José Miguel Pereira

**Affiliations:** Centro Hospitalar de São João, Alameda Prof. Hernâni Monteiro, 4200-319 Porto, Portugal

**Keywords:** Smoking, Emphysema, Bronchitis, Interstitial lung diseases, Fibrosis

## Abstract

Cigarettes are well-recognized risk factors responsible for the emergence of a variety of pathologic conditions affecting both the airways and the lungs. Smoking-related lung diseases can be classified as chronic obstructive pulmonary disease (COPD) and several types of interstitial diseases, such as pulmonary Langerhans cell histiocytosis, bronchiolitis, desquamative interstitial pneumonitis, acute eosinophilic pneumonia, and interstitial fibrosing lung diseases. The evidence of combined lower lung fibrosis and predominant upper lung emphysema is renowned as a distinct clinical entity, named combined pulmonary fibrosis and emphysema. Although computerized tomography permits an adequate classification and distinction of these diseases, the clinical, imaging, and histological features often overlap and coexist in a single patient. Therefore, a combined radiologic and pathologic approach, in the appropriate clinical setting, is useful for best comprehension and distinction of these entities. Our goals are to describe the imaging features in smoking-related lung diseases and how the pathological manifestations translate on high-resolution computerized tomography.

## Key points

- COPD is one of the most common smoke-related causes of death, depicted by the spirometric evidence of irreversible and usually progressive airflow limitation. It includes chronic bronchitis and emphysema. The peripheral and smaller airways are also affected in both number and caliber.
- Changes in smoking habits are known inducers of the development of acute eosinophilic pneumonia (AEP): initiation of smoking habit, resumption after interruption, and increased frequency of smoking.
- Pulmonary Langerhans cell histiocytosis (PLCH) is an uncommon disease virtually exclusive of smokers. Over time, the cellular nodules are replaced by polymorphic fibrotic scars associated with distorted and enlarged air spaces.
- Besides the difference of imaging findings, desquamative interstitial pneumonia (DIP) and respiratory bronchiolitis/respiratory bronchiolitis-interstitial lung disease (RB/RB-ILD) are a spectrum of the same pathologic event, characterized by the excess of macrophages in the distal airways.
- Fibrosis is also a common radiological feature ranging from sparse fibrosis along the alveolar walls, termed AEF, to a pattern of diffuse interstitial fibrosis, which can represent usual interstitial pneumonia (UIP) in some cases.

## Introduction

Cigarettes are a noxious mixture containing around 5000 chemicals and considered as one of the most important causes of chemically mediated disorders in humans. Both direct toxicity and induced immune-mediated response lead to both reversible and irreversible damage from central airways to most distal airways and lung parenchyma [1–3].

The most common smoking-related causes of death include numerous types of cancer, particularly lung cancer, and chronic obstructive pulmonary disease (COPD). Adding to these disorders, cigarettes are a well-known etiologic factor linked with the development of some types of interstitial lung disease (ILD), namely AEP, DIP, RB-ILD, and PLCH. Smoking is also responsible for the development of fibrotic lung disease [4–7]. These lung diseases related to smoking are a spectrum of the same pathologic process. Pathologists usually find a mixture of histopathological patterns and a single diagnosis is often difficult to make [5]. Although the majority of the smokers have a certain degree of inflammatory changes in the airways, just a subgroup of individuals develops clinically relevant respiratory disease. Both genetic and exogenous triggers, such as allergens or infections, may be implicated in the development of the disease [7].

In this article, we describe and illustrate the characteristic clinical features, imaging findings, and pathologic findings of diffuse lung diseases related to smoking, encompassing COPD and ILDs. We emphasize the need of a multidisciplinary approach (clinical, radiological, and pathological) for better comprehension and distinction of these entities.

## Discussion

### Chronic obstructive pulmonary disease

COPD is depicted by the spirometric evidence of irreversible and usually progressive airflow limitation. The disease comprehends distinct however overlapping obstructive disorders, such as chronic bronchitis, emphysema, and also affecting the distal airways of the lung, with both reductions in the number and the caliber. Bronchiolitis is the earliest lesion in COPD, with narrowing and loss of terminal bronchioles preceding emphysematous changes [3, 8]. Emphysema results from permanent enlargement and wall destruction of the airways distal to the surviving terminal bronchioles, progressing in severe cases to coalescence of destroyed lobules [8, 9]. Centrilobular and panlobular emphysema have clinical significance, often associated with increased dyspnea and poorer functional capacity. Additionally, centrilobular emphysema is related to smoking habits, and panlobular emphysema is associated with low body mass index (BMI). Paraseptal emphysema is more common in men and is frequently of little physiologic significance, except for the development of pneumothorax secondary to the presence of paraseptal bleb/bulla [10]. Visual CT evaluation is considered the clinical gold standard for the assessment and characterization of centrilobular and panlobular emphysema, also demonstrated to be valid regarding the pathologic assessments [10–14].

**Fig. 1:**
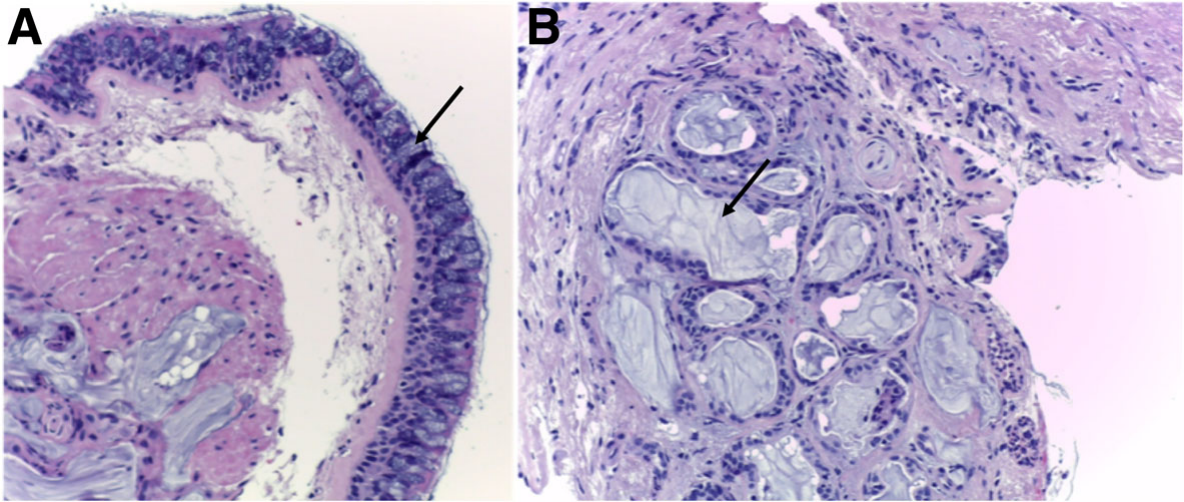
Chronic bronchitis. **a** and **b** Low-power hematoxylin and eosin stain (H&E) shows an increased number of goblet cells (arrow in **a**) and hyperplasia of submucosal glands (arrow in **b**). Hypersecretion of mucus from goblet cells is also visualized

**Fig. 2:**
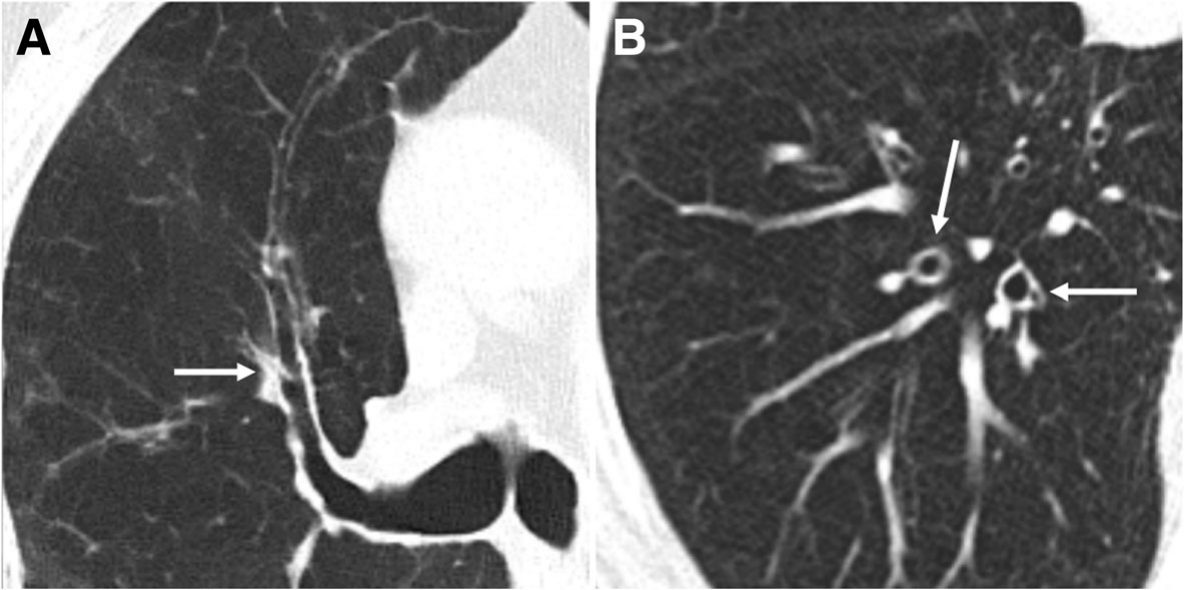
Bronchial wall thickening in a 79-year-old heavy smoker man. **a** and **b** Axial images from a chest CT show thickening of the bronchial walls (arrows). Bronchial wall thickening is an important predictor factor of FEV1 and of the risk of acute exacerbation

*Chronic bronchitis* consists in the presence of inflammation in the large airways. Clinically, patients present with chronic cough and sputum during at least 3 months per year in two consecutive years [15]. Chronic bronchitis is triggered and sustained by the activation of an abnormal immune response due to long-term cigarette smoking, causing overproduction of mucus from goblet cells, thickening, and fibrosis of the bronchial walls (Fig. 1). The condition promotes a further reduction of the caliber of the airways, predisposing expiratory collapse. Imaging depicts wall thickening of the large airways, and endobronchial mucus plugging may also be depicted (Fig. 2) [15, 16]. Bronchial wall thickening is consistently associated with a decline in FEV1 and with the risk of acute exacerbation and hospitalization [16–19].

**Fig. 3:**
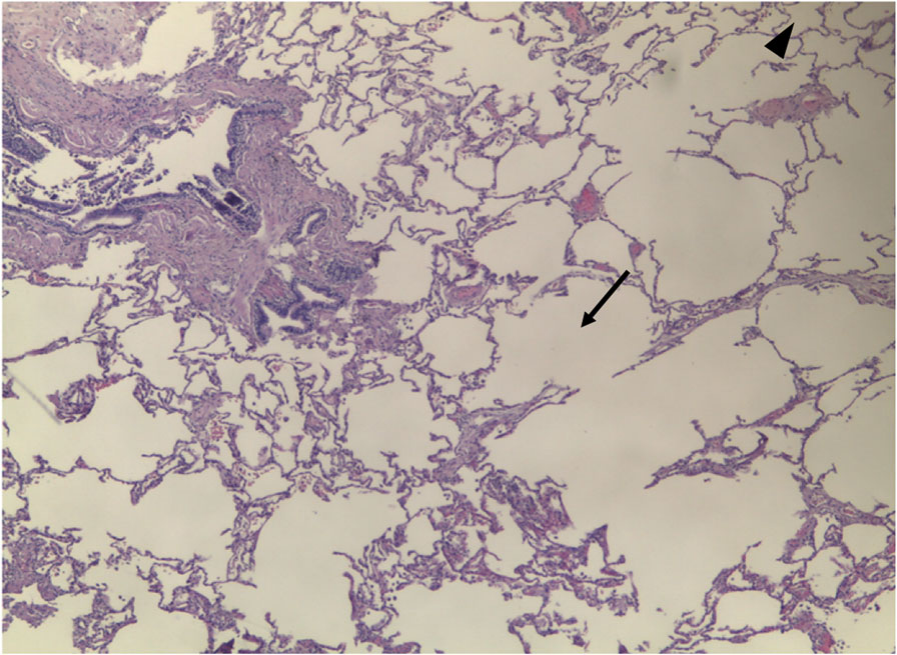
Centrilobular emphysema. Low-power hematoxylin and eosin (H&E) stain shows emphysematous spaces surrounding the terminal bronchioles (arrow; arrowhead in normal parenchyma). Abnormally large airspaces are the result of the destruction of alveolar walls

**Fig. 4:**
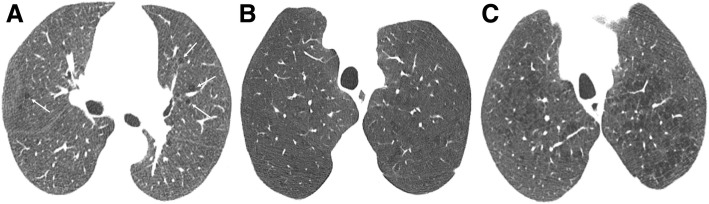
Subtypes of CLE according to the Fleischner Society guidelines on HRCT. Axial images in 3 smokers show **a** trace CLE, with minimal centrilobular lucencies occupying less than 0.5% of a lung zone (arrows); **b** mild CLE, defined as scattered centrilobular lucencies affecting 0.5% to 5% of a lung zone; and **c** moderate CLE, with numerous centrilobular lucencies occupying more than 5% of any lung zone

**Fig. 5:**
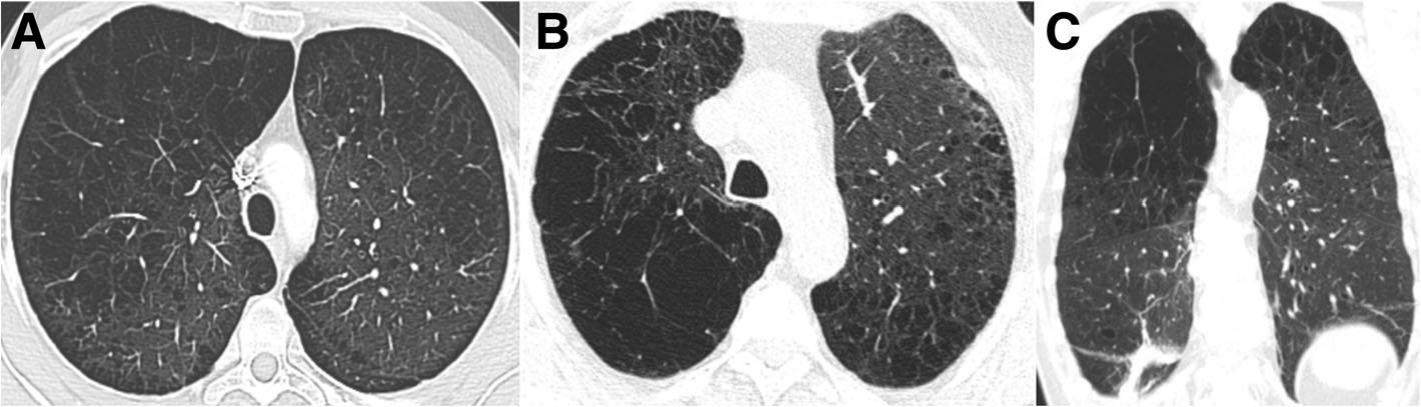
Subtypes of severe CLE according to the Fleischner Society guidelines on HRCT. **a** Axial CT image depicts confluent CLE with coalescent centrilobular lucencies without significant distortion of the pulmonary architecture. **b** Axial and **c** coronal images show ADE, with panlobular lucencies, distortion of the underlying pulmonary architecture, and hyperexpansion of the lung

*Centrilobular emphysema* (CLE) results from an abnormal dilatation of the airspaces distal to the terminal bronchioles. This subtype of emphysema is strongly related to cigarette smoking with higher airway inflammatory cell content and usually with upper lung predominance [10]. CLE originates from the destruction and dilation of bronchioles, with further coalescence of several primary lesions (Fig. 3). On imaging, small well or poorly demarcated regions of low attenuation can be depicted, surrounded by areas of the normal lung [3, 8–10]. Fleischner Society’s guideline scoring of CLE [20] characterizes emphysema as trace when involving less than 0.5% of a lung region, mild when concerning 0.5–5%, and moderate if more than 5% (Fig. 4). Severe emphysema is classified as confluent (coalescence of centrilobular lucencies) or advanced destructive emphysema (ADE) if the expansion of the entire secondary lobule, distortion of the pulmonary architecture, and splaying or decreased caliber of vessels are present (Fig. 5).

**Fig. 6:**
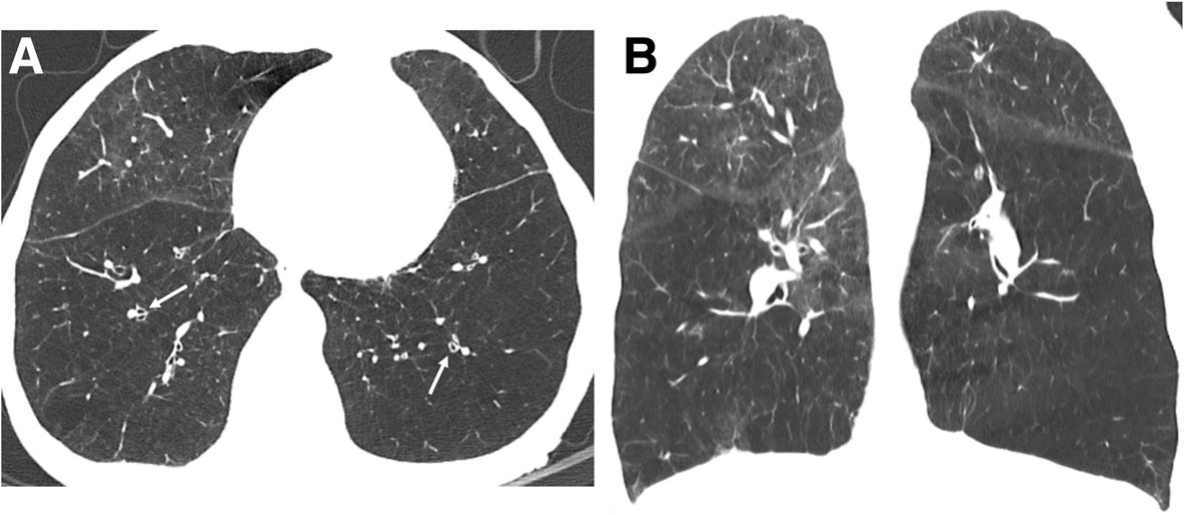
PLE in a 51-year-old man with alpha-1 antitrypsin deficiency. **a** Axial and **b** coronal CT images show hyperinflation and lower lobe predominant emphysema, involving the entire secondary pulmonary lobules. Bronquial wall thickening is also depicted (arrows)

*Panlobular emphysema* (PLE) like ADE indicates destruction across the lobule. This type of emphysema presents in a younger age group (30–44 years of age). The findings are lower lung predominant in about two-thirds of the individuals (Fig. 6) [3, 8–10, 20]. It is commonly linked to a mutation in the alpha 1-antitrypsin gene, in which Z allele accounts for approximately 95% of clinically recognized cases, causing unopposed action of neutrophil elastase with consequent destruction of lung parenchyma [21].

**Fig. 7:**
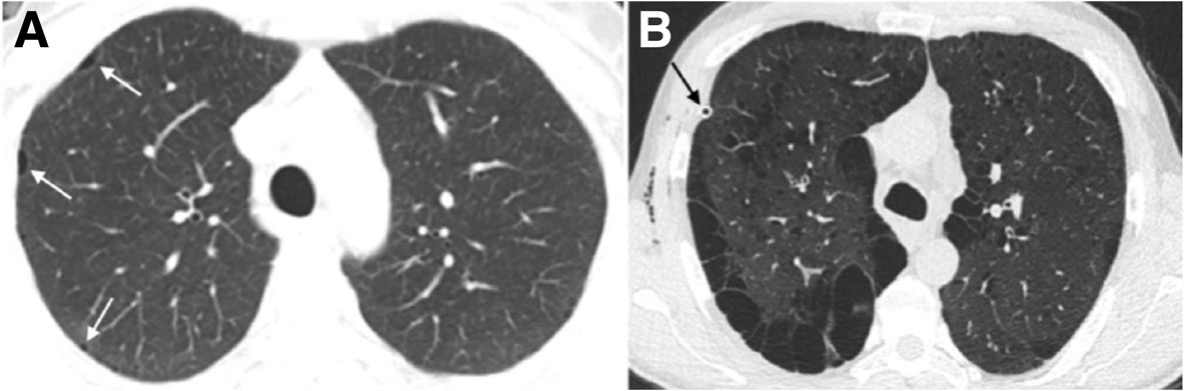
Subtypes of PSE according to the Fleischner Society guidelines on HRCT. **a** Axial CT shows mild PSE (white arrows) with up to 1 cm juxtapleural well-demarcated lucencies. **b** Axial CT depicts substantial PSE, defined as cyst-like lucencies or bullae greater than 1 cm adjacent to the pleura. The patient had spontaneous pneumothorax and black arrow depicts a chest drain. Subcutaneous emphysema is also visible in the right thoracic wall

**Fig. 8:**
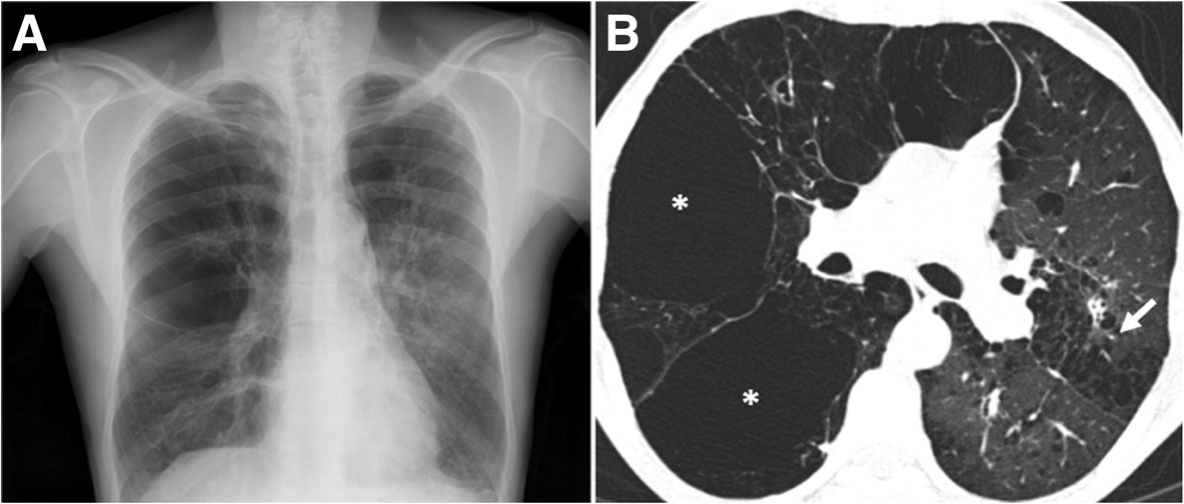
Bullous emphysema in a 55-year-old man. **a** Posteroanterior (PA) radiograph shows lucencies in the superior half of the right hemithorax and in the upper left hemithorax. **b** Axial CT shows upper lobe predominant bullae (asterisks) in the subpleural surface of the right lung. Moderate CLE is also present (arrow)

*Paraseptal emphysema* (PSE) is secondary to emphysematous changes of the distal acinus, adjacent to the visceral pleura, including fissures. PSE is also scored as mild (up to 1 cm juxtapleural well-demarcated lucencies) or substantial (cyst-like lucencies or bullae greater than 1 cm adjacent to the pleura) (Fig. 7). Bullae are a risk factor for spontaneous pneumothorax, and they can also be large enough to cause severe compression and reduction of the pulmonary function of the remaining lung (Fig. 8) [20].

### Acute eosinophilic pneumonia

AEP is an uncommon condition that occurs most frequently in males between the second and forth decades of life. Approximately two-thirds of patients have smoking habits [22–25]. Changes in smoking habits are known inducers of the development of AEP: initiation of smoking habit, resumption after interruption, and increased frequency of smoking [26–28]. The clinical presentation is generally unspecific, letting the disease be frequently misdiagnosed as other most common entities such as community-acquired pneumonia. The clinical symptoms are acute, with duration of the respiratory illness of less than a month, and characterized by moderate fever, cough, dyspnea, pleuritic pain, malaise, myalgia, and night sweats. Acute respiratory failure is frequent and mechanical ventilation is often required [22, 25]. Therefore, the disease is severe, and most patients fulfill diagnostic criteria for acute lung injury (ALI) or acute respiratory distress syndrome (ARDS). The key to diagnosis is evidence of eosinophilia in the bronchoalveolar lavage (BAL), with more than 25% of eosinophils on the cell count. Nevertheless, blood eosinophils are frequently at normal levels at the beginning of the clinical presentation and may rise after a few days. BAL is sterile with no bacterial growth during the disease course [22, 23, 25].

Lung biopsy is usually not needed to meet the diagnosis. It shows alveolar and interstitial eosinophilia and alveolar damage. Additional features include nonnecrotizing perivascular inflammation, eosinophilic abscesses, interstitial lymphocytes, organizing fibrinous exudate in the alveoli, type II pneumocyte hyperplasia, and also involvement of the airway [22, 29].

**Fig. 9:**
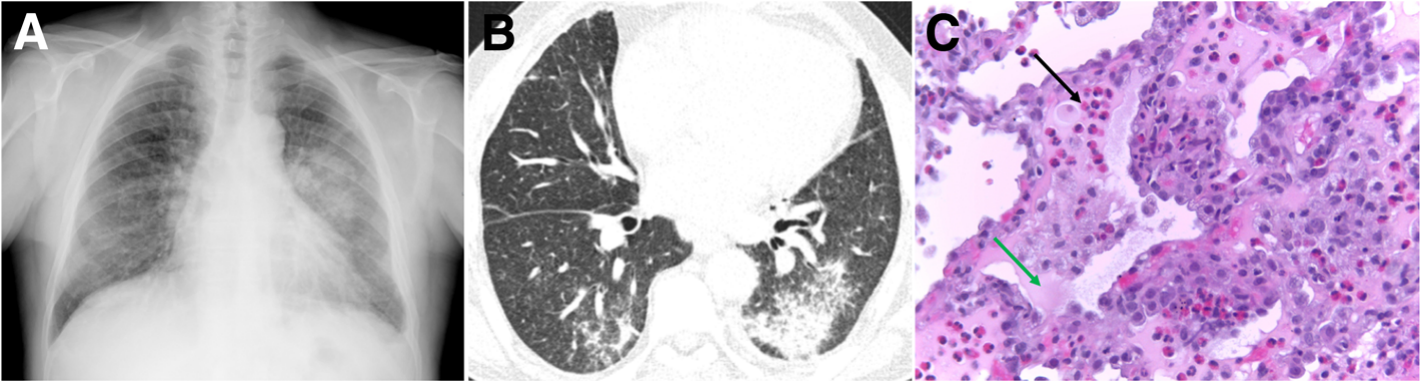
Radiologic-pathologic correlation of AEP. **a** Posteroanterior (PA) radiograph shows bilateral ground-glass opacities and consolidation in the mid-left lung zone. A small left pleural effusion is present. **b** Axial CT shows peripheral lower lobe consolidations and ground-glass opacities, mainly on the left. **c** Transthoracic lung biopsy showing eosinophils (black arrow) infiltrating the interstitium and the alveolar spaces; edema (green arrow) and reactive pneumocytes are seen; no necrotizing vasculitis is observed (H&E, × 400)

Imaging findings are predominant in the lower lungs, showing diffuse consolidations, ground-glass opacities (GGO), ill-defined centrilobular nodules, smooth septal thickening, and unilateral or bilateral pleural effusion (Fig. 9). The radiologic differential includes infection, fluid overload, ALI/ARDS, hypersensitivity to drugs, and pulmonary hemorrhage [7, 24, 25, 29, 30].

Treatment with steroids achieves an excellent response, with resolution within days of the immunologic process [22, 23].

### Pulmonary Langerhans cell histiocytosis

PLCH is an unusual respiratory disease found most commonly in young adults between the third and fourth decades of life. It is exceedingly rare in children and occurs in most cases as a part of disseminated LCH secondary to an abnormal immune response. Over 95% of PLCH patients are smokers and it is estimated that the disease affects approximately 3–4% of smokers. PLCH affects both genders equally [31–33]. Patients usually report fatigue, weight loss, exertional dyspnea, and non-productive cough. Pneumothorax may be the first sign in 15–20% of patients. Although, approximately 20% of patients reported no symptoms at the time of disease detection [33].

Histopathologic findings reveal bronchiole-centered, stellate nodules, containing Langerhans cells, and interspersed with other inflammatory cells (lymphocytes, macrophages, monocytes). Langerhans cells are quite large cells, containing pale cytoplasms and convoluted nucleus, which resemble coffee grains. They are immunoreactive with CD1a and S-100. Along the disease course, cellular nodules evolve from mixed cellular and fibrotic nodules to entirely polymorphic scars associated with enlarged and distorted air spaces [32–38].

**Fig. 10:**
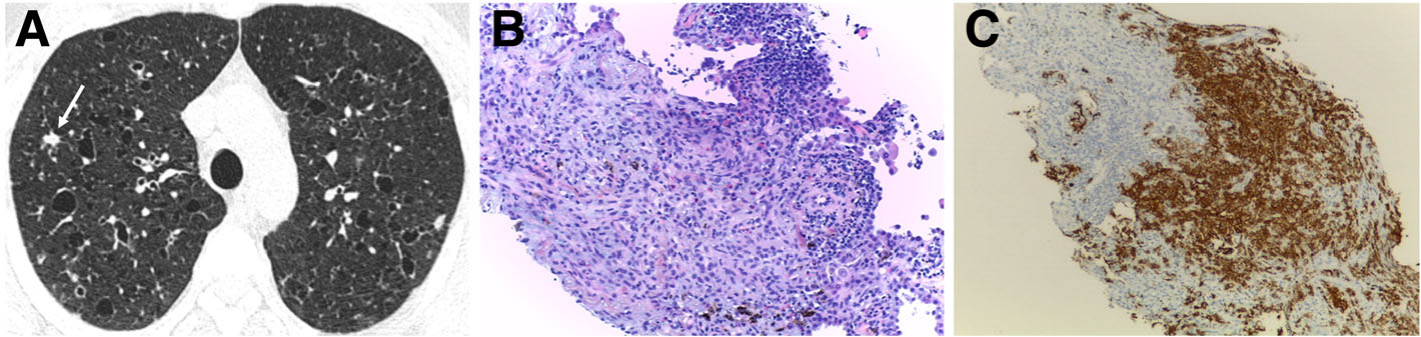
Pulmonary Langerhans cell histiocytosis in a 40-year-old man. **a** Axial CT image shows upper lobe predominance of nodules and cysts of varying wall thickness and irregular margins. Arrow depicts a stellate cellular nodule. **b** Transthoracic lung biopsy of the nodule highlighted in **a** shows a nodular aggregate of cells (lymphocytes, eosinophils, plasma cells, and Langerhans cells) centered in bronchioles (arrow) and extending to the interstitium (H&E, × 200). **c** CD1a immunostaining highlights the Langerhans cells (CD1a, × 100)

**Fig. 11:**
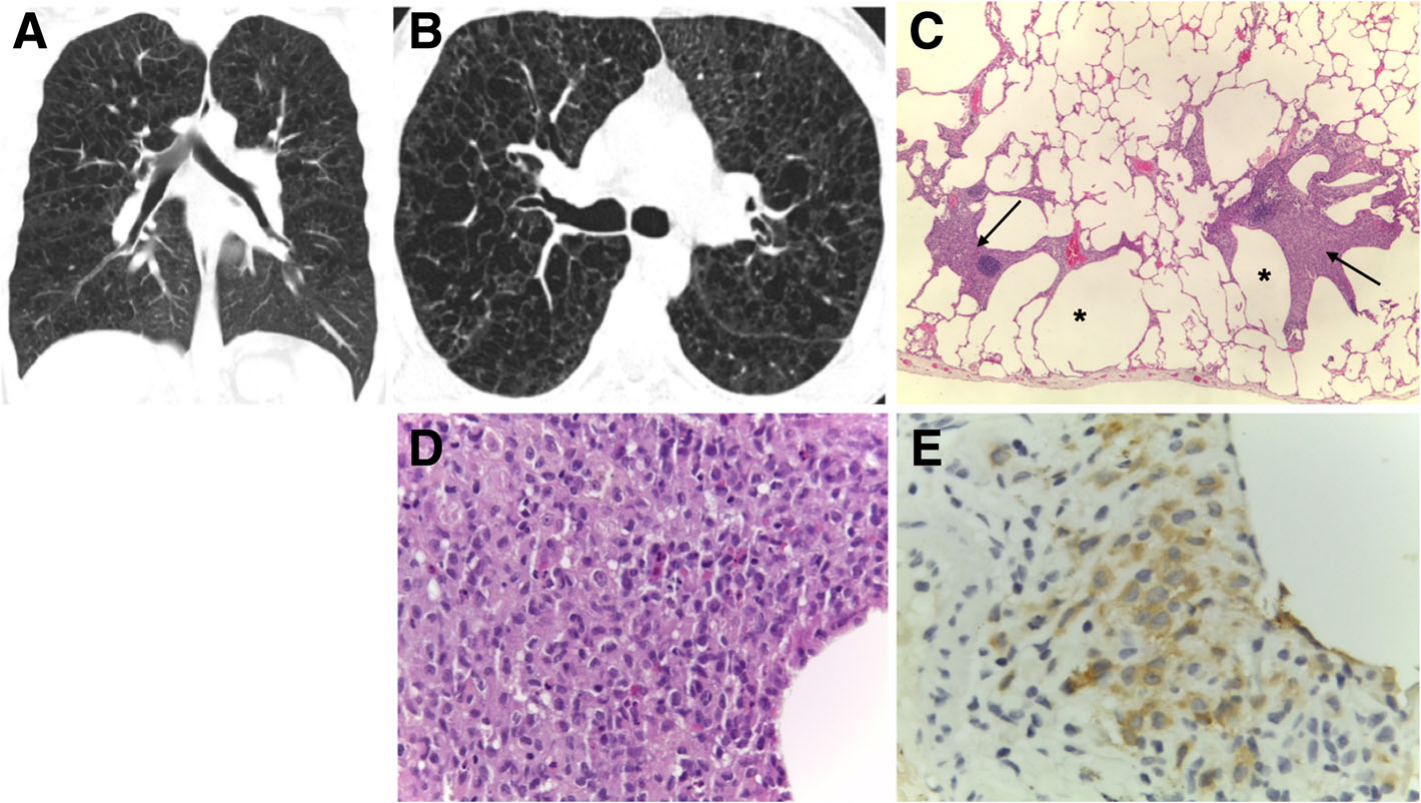
PLCH. **a** Coronal and **b** axial CT images in a 30-year-old man show diffuse upper lobe predominant lucencies with basilar sparing. Imaging findings were thought to represent severe confluent emphysema. **c** Small cysts (asterisk) with nodular aggregates of Langerhans cells in their walls, with a stellate appearance and centered in bronchioles (arrows) (H&E, × 40). **d** The cells in the nodules have vesicular nuclei and a pale cytoplasm and sometimes with a reniform appearance; some eosinophils are present (H&E, × 400). **e** CD1a immunostaining highlights the Langerhans cells

**Fig. 12:**
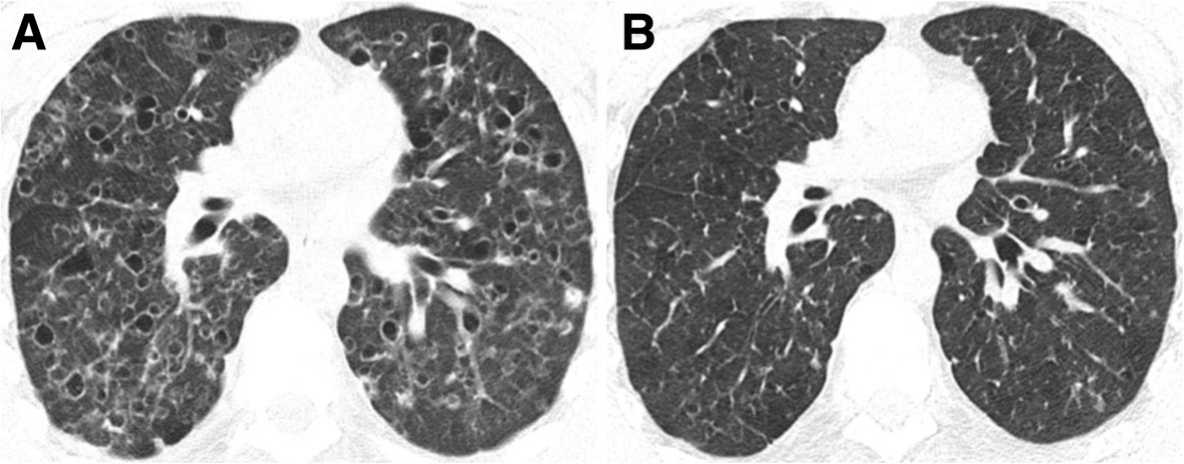
PLCH. Regression of the imaging findings over time (2-year interval), after smoking cessation. **a** Axial CT image showing cysts with different shapes and sizes diffusely distributed in both lungs. A few cellular and mixed nodules are also present. **b** Axial CT image at 2-year interval showing almost complete resolution of the imagiological findings

**Fig. 13:**
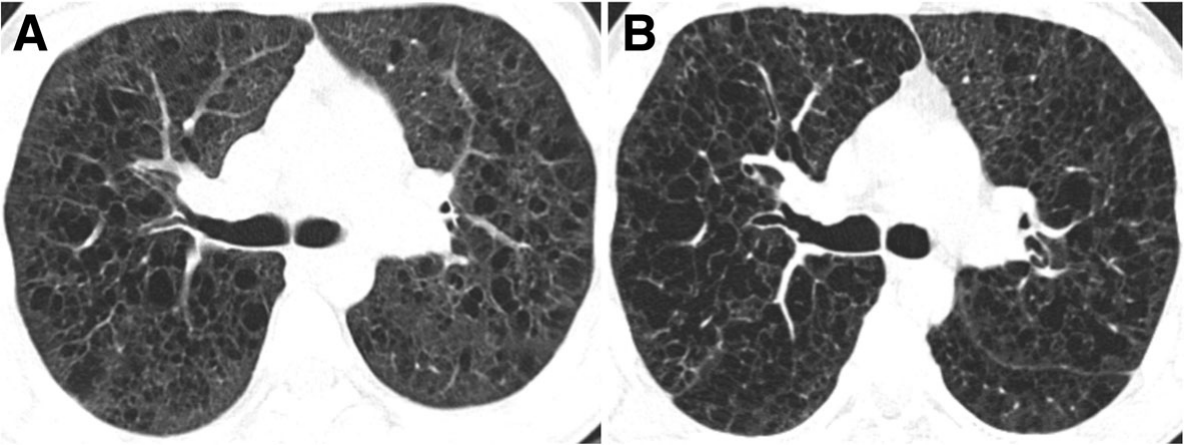
PLCH. Progression of the imaging findings over time (10-year interval), despite smoking cessation, in a 30-year-old man. **a** Axial CT image showing diffuse bizarre cysts with walls of varying thickness. **b** Axial CT image at 10-year interval showing progression of the number and size of the cysts in both lungs

Imaging reveals upper and middle lung predominance of nodules and cysts with variable wall thickness and irregular margins. The disease characteristically spares the costophrenic sulci, extreme apices, and part of the middle lobe and lingula. Imaging findings have a typical progression over time from a predominant nodular pattern to diffusely distributed cysts with bizarre shapes (Fig. 10). The later disease stage is associated with significant emphysematous areas either related to PLCH scars or usual emphysema due to smoking (Fig. 11). GGOs are a frequent imaging finding and may be associated with the presence of other smoke-related diseases, for example, RB and DIP [6, 7, 24, 29, 30, 33, 38]. Smoking cessation is a fundamental key in the treatment process of PLCH. After smoking cessation, symptoms and radiologic alterations may partially regress or stabilize in more than half of the patients (Fig. 12) [34, 39–46]. However, one third to a half of the patients may show respiratory failure and disease progression, despite smoking cessation (Fig. 13) [47].

**Fig. 14:**
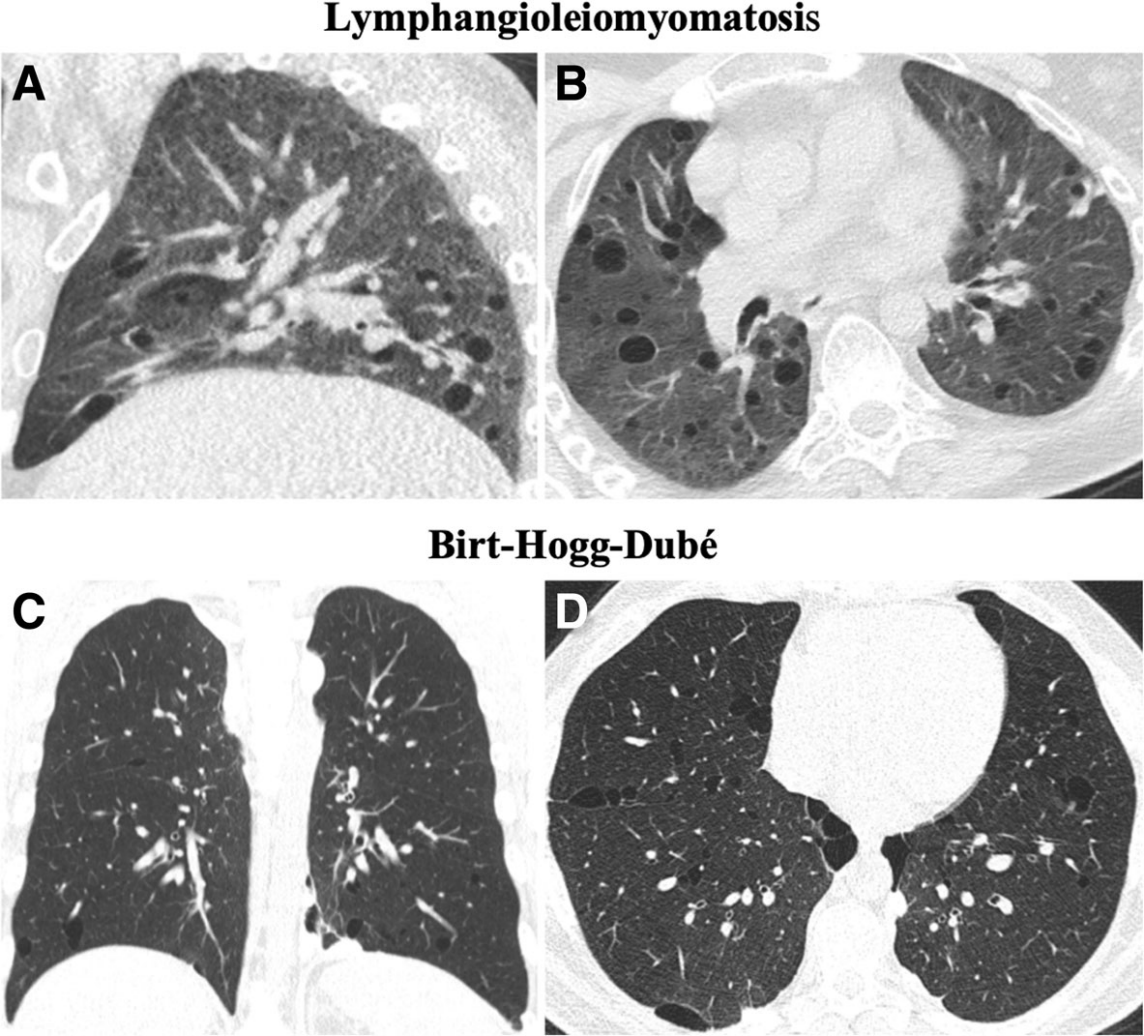
Differential diagnosis of PLCH. **a** Sagittal and **b** axial CT images show a 50-year-old woman with LAM. There are multiple rounded-shaped cysts, with a relatively uniform distribution with no zonal predilection and affecting the lung bases. **c** Coronal and **d** axial CT images depict multiple cysts with a lower zone distribution, in a 35-year-old male with Birt-Hogg-Dubé syndrome. The patient had a history of recurrent pneumothoraces

PLCH should be differentiated from lymphangioleiomyomatosis (LAM), in which cysts tend to have more regular and rounded shapes and more uniformly distribution through the lung. Other differentials should also be considered, such as pulmonary metastasis, sarcoidosis, Birt-Hogg-Dubé syndrome, and infections (Fig. 14) [32, 35, 48, 49].

### Respiratory bronchiolitis and desquamative interstitial pneumonia

RB-ILD and DIP are moderately uncommon diseases related to smoking. On clinical presentation, patients usually complain about insidious dyspnea and cough over the course of weeks to months [5–7, 50–54]. The clinical disease course of RB-ILD and DIP tends to be stable in most of the patients. However, the rates of impairment are worse in those with DIP, in which diffusing capacity for carbon monoxide (DLco) may be severely reduced. As a consequence, deaths can occur in patients with DIP, but there are no described related deaths in those with RB-ILD [5, 50, 51]. RB is a classic histological marker of smoking, encountered in the lungs of nearly all active smokers. However, patients with RB are fundamentally asymptomatic, and the disease does not portend any clinical significance. RB and RB-ILD are differentiated by each other by the presence of respiratory symptoms and abnormal pulmonary function tests in the last one [5–7, 50, 51].

Histopathologic examinations of RB and DIP show an excess number of pigmented or smokers’ macrophages involving the distal airways and peribronchiolar airspaces. However, DIP presents with pigmented macrophages diffusely filling the alveoli, with associated thickening of the septa secondary to the presence of inflammatory cells. On DIP findings, the degree of interstitial fibrosis is usually mild and more severe than in RB. Both entities represent a severity spectrum of the same pathologic event, with an excess number of macrophages filling the distal airways and alveoli secondary to an immune-mediated response due to smoking [5, 24, 29, 51, 55, 56].

**Fig. 15:**
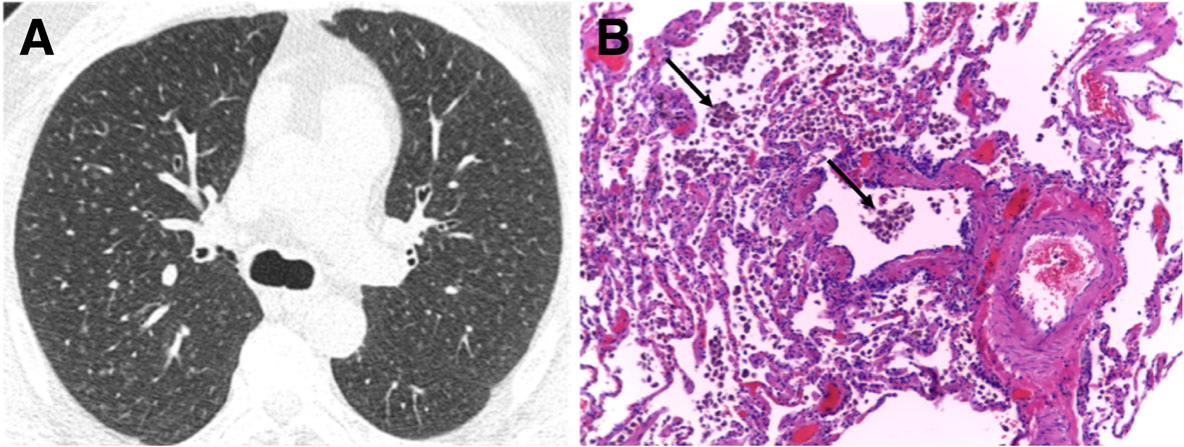
RB-ILD. **a** Axial CT image depicts diffuse, ill-defined centrilobular nodules. Due to deteriorating symptoms, the patient underwent lung biopsy. **b** Excess numbers of alveolar macrophages (arrows) with light brown granules filling the cytoplasm, in a predominant centriacinar location; mild interstitial lymphocytic infiltrate (H&E, × 100)

**Fig. 16:**
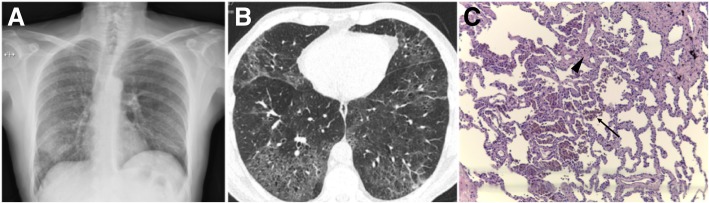
DIP in a 58-year-old heavy-smoker man. **a** Posteroanterior (PA) radiograph shows reticular opacities and ground-glass attenuation in both lungs, predominantly in the lower lung zones. **b** Axial CT image shows ground-glass opacities with cystic changes in the lower and mid lobes and lingula. Images show well-defined areas of sparing, creating a mosaic attenuation. Mild areas of reticulation and bronchiectasis are also present, signifying fibrosis. **c** Large numbers of alveolar macrophages with light brown granules filling the cytoplasm (arrow); mild interstitial lymphocytic infiltrate and mild fibrosis (arrowhead) (H&E, × 200)

On imaging, RB/RB-ILD shows upper lung predominance of the findings, characterized by the evidence of low attenuation centriacinar nodules, GGOs, bronchial wall thickening, few thickened interlobular septa, and also lobular air-trapping, especially depicted on expiratory CT. These findings are generally associated with emphysema (Fig. 15). The radiological findings in DIP are lower lobe predominant and characterized by GGOs and reticular opacities interposed with relatively normal lung zones, forming a mosaic attenuation. Small cystic spaces may be depicted in the areas of GGO, representing dilated alveolar ducts or centrilobular emphysema. The distribution of findings is more often subpleural but may also be random or diffuse (Fig. 16) [6, 7, 24, 29, 30, 54, 56]. Associated findings of RB and emphysema are frequently visualized. The evidence of areas of mild pulmonary fibrosis in DIP, characterized by increased septal lines, traction bronchiectasis, and lung distortion, is associated with disease progression in some individuals, despite smoking cessation and steroids [53].

**Fig. 17:**
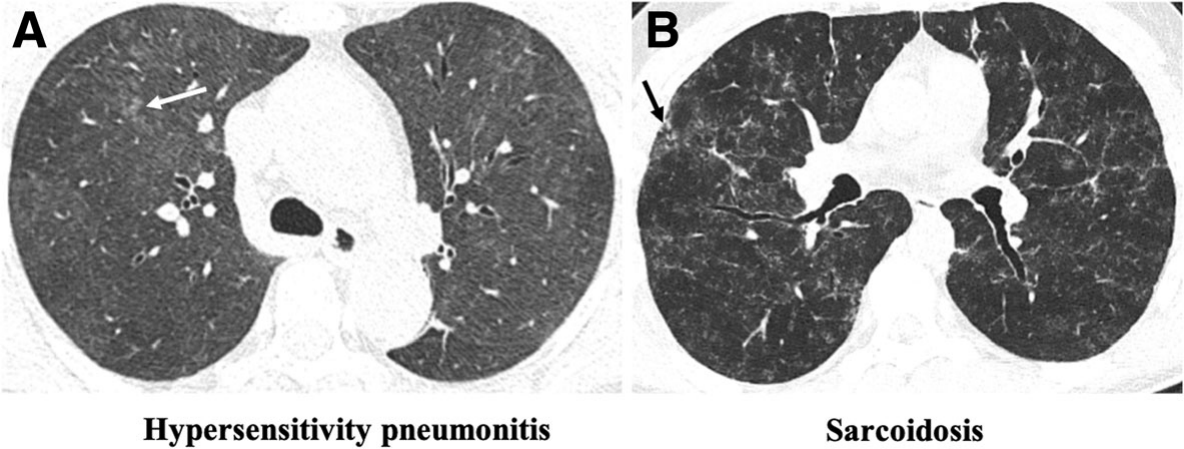
Differential diagnosis of RB-ILD. **a** Axial CT image depicts subacute hypersensitivity pneumonitis (HP) in a 71-year-old woman with a long history of exposition to moldy hay (farmer’s lung). Imaging findings of HP are similar to RB-ILD, which also appears as centrilobular nodules predominantly in the upper lobes (arrow) and patchy areas of ground-glass attenuation. A clinical history is crucial to make the differential diagnosis. **b** Sarcoidosis in a 37-year-old man. Axial CT image shows diffuse areas of nodularity with peribronchial distribution and also in relation to the subpleural region (arrow). There are also some surrounding ground-glass opacities

**Fig. 18:**
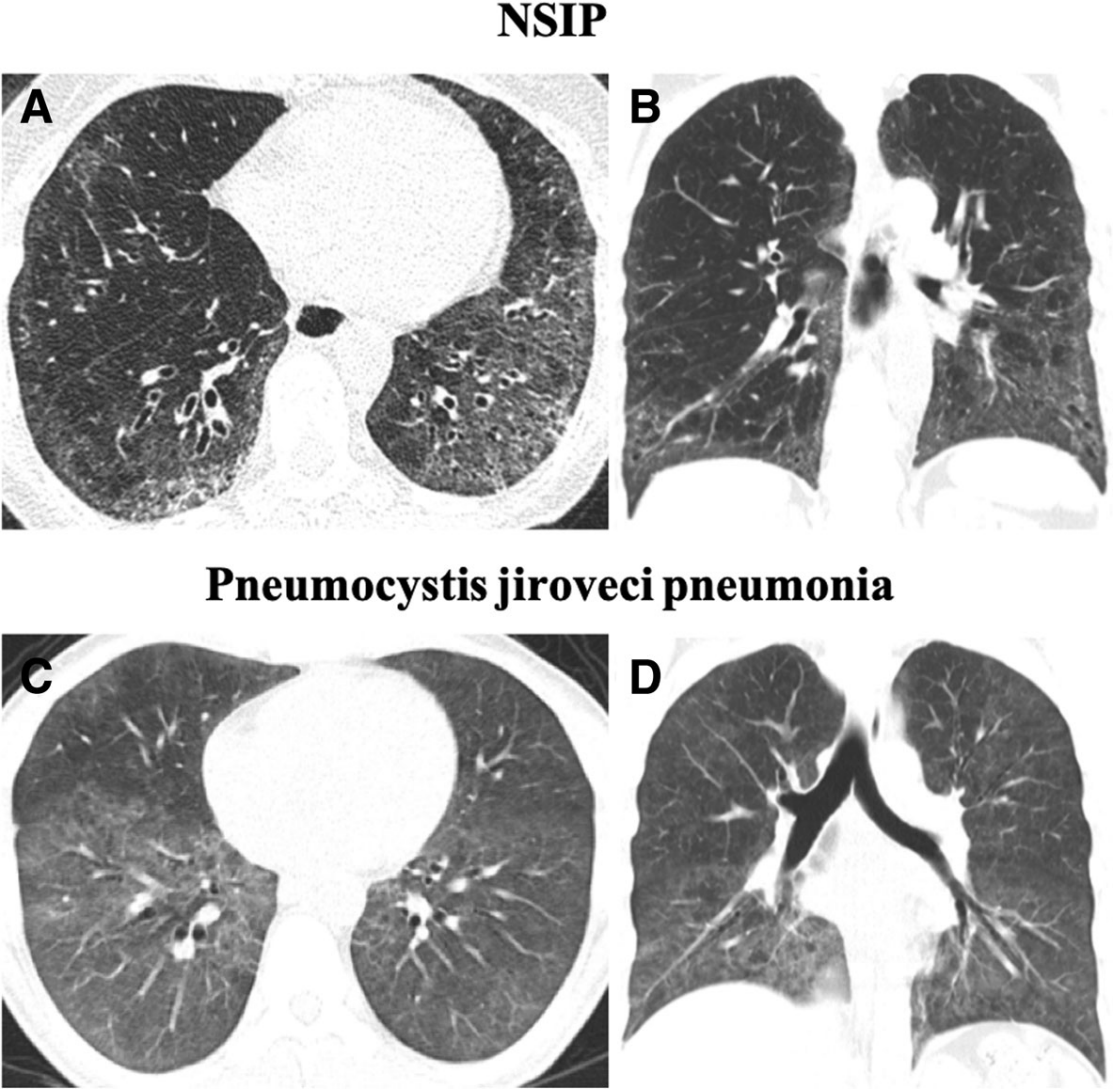
Differential diagnosis of DIP. **a** Axial and **b** coronal CT images depict NSIP in a female patient with systemic sclerosis. CT findings show bilateral and asymmetrical ground-glass opacities, with a lower lobe predominance and immediate subpleural sparing. **c** Axial and **d** coronal CT images depict pneumocystosis in an immunocompromised patient with HIV. Imaging findings show bilateral ground-glass opacities, predominantly in the lower lobes

Some differential diagnoses are important to be considered in association with patient’s complete clinical history, including NSIP, hypersensitivity pneumonitis, and atypical infections, including pneumocystosis (Figs. 17 and 18) [7, 24, 29, 50].

### Interstitial fibrosis

#### Airspace enlargement with fibrosis/smoking-related interstitial fibrosis

Cigarette smoke leads to alveolar wall fibrosis that increases with time and intensity of exposure, called AEF, also termed as SRIF. Pulmonary fibrosis severity ranges from sparse fibrosis in the alveolar walls to diffuse fibrosis, with dense, paucicellular, eosinophilic collagen that has a waxy quality on pathologic examination. Hypertrophic smooth muscle bundles can also be depicted and perhaps predominate. AEF is also accompanied by features of emphysema and RB [57–59]. The fibrosis is confined in the subpleural and peribronchiolar interstitium, with relative preservation of the lung architecture ^60^. It is important to mention that AEF is an incidental histologic or radiological finding characterized by interstitial fibrosis exceeding the fine fibrosis often seen in emphysema alone. Most patients have stable disease and good survival time [60, 61]. Therefore, patients with progressively worsening exertional dyspnea and cough might have AEF accompanied by chronic interstitial pneumonia such as UIP or nonspecific interstitial pneumonia (NSIP). In these cases, lung biopsy is crucial for definitive diagnosis [62].

**Fig. 19:**
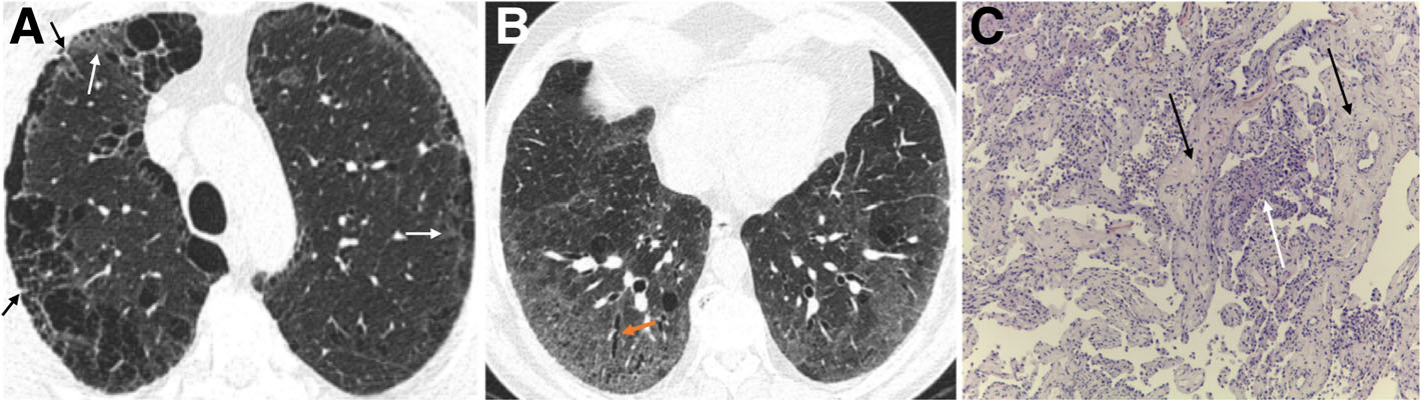
AEF and DIP combined with fibrotic NSIP pattern. **a** Axial CT image through the upper lobes shows CLE and PLE, associated with reticulation (black arrows) and discrete patchy ground-glass opacities (white arrows). The areas of emphysema within the areas of GGO appear to have more well-defined walls, a finding termed as AEF. **b** Axial CT at the lower lobes shows GGO with scattered cystic changes, mild reticulation, and bronchiectasis (orange arrow), signifying underlying fibrosis. **c** Lung transbronchial criobiopsy with uniform thickening of alveolar septa by collagen deposition (black arrows), mild associated inflammation, emphysema, and respiratory bronchiolitis (white arrow) (H&E, × 100)

Radiological features of AEF are subpleural sparing thin-walled cysts (TWCs), associated with reticular and ground-glass opacities. Imaging distinction between the typical honeycombing present in idiopathic pulmonary fibrosis (IPF) and TWCs related to smoking may be confusing. The cysts in AEF appear as thin-walled cysts (less than 1 mm) predominantly distributed in the upper lobes and upper and middle portion of the lower lobes, slightly distant from pleura, affecting deeper lung parenchyma (Fig. 19a) [63–65].

#### NSIP/UIP

The progression of fibrosis leads to increasing lower-lobe predominant GGOs, traction bronchiectasis, and reticulation, in a pattern compatible with NSIP on imaging examination (Fig. 19) [5, 24, 61, 66]. These changes are often accompanied by other smoking-related findings, such as emphysema, DIP, and RB. In AEF, emphysema may appear better-demarcated secondary to the presence of fibrosis in the alveolar walls [5, 63–65].

IPF is a progressive chronic fibrosing lung disease with unknown etiology and pathologically described by a pattern consistent with UIP [67]. It represents the most common but also the most severe type of ILD, affecting most frequently males over the age of 65 years. The median survival time after the diagnosis ranges from 2 to 4 years. Cigarette smoking is considered a probable risk factor for the development of IPF, with an odds ratio ranging from 1.6 to 2.9 [50, 67–69]. Cigarette smoking is also linked to lower survival time, in relation to non-smoker IPF patients [70, 71]. Imaging features are often bilateral and asymmetric and include peripheral and basal predominant traction bronchiectasis, reticular opacities, and honeycombing, with minimal GGOs. Pulmonary volumes are typically low. The findings are spatially and temporally heterogeneous, with areas of varying disease extent and severity adjacent to regions of a more normal lung. The evidence of any of the following features should prompt an alternative diagnosis: fibrosis predominantly located in the upper or mid lungs, extensive GGOs, peribronchovascular distribution, diffuse cysts or nodules, predominant consolidation, and presence of air trapping with substantial mosaic attenuation [72].

#### Combined pulmonary fibrosis and emphysema

The disease termed CPFE is a severe respiratory condition integrating imaging features of both pulmonary fibrosis and emphysema. Patients present with similar symptoms to IPF and emphysema; however, they have relatively preserved spirometry, as a result of the combined obstructive disease in distal airways and restrictive fibrosis. The clue for the diagnosis is the evidence of the physiologic decline in diffusing capacity and characteristic CT features [73–79].

**Fig. 20:**
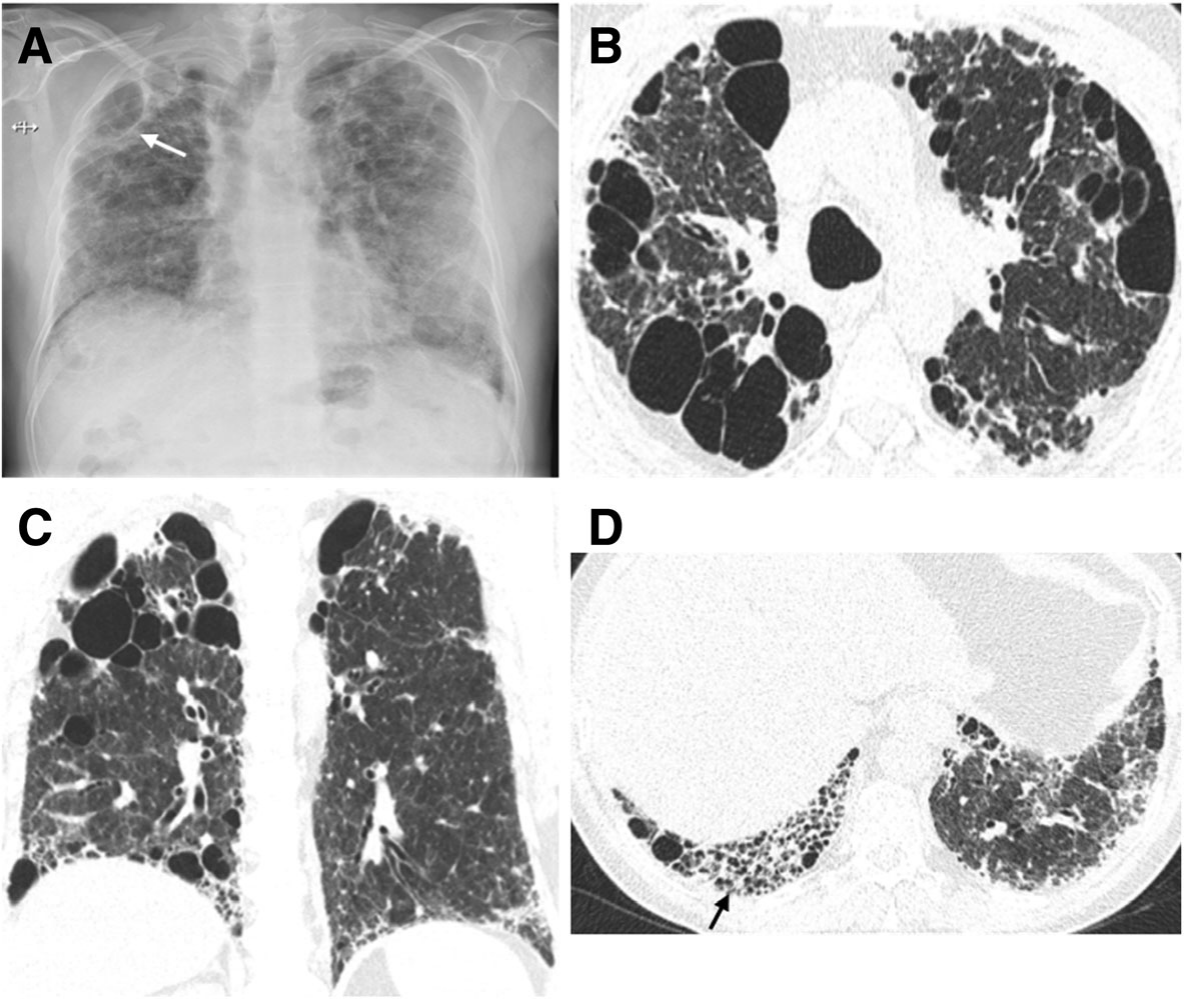
Combined pulmonary fibrosis and emphysema (CPFE). **a** Posteroanterior (PA) radiograph shows right volume loss, diffuse bilateral reticular opacities, and GGO. There are lucencies in the upper lobes (white arrow), signifying bullous emphysema. **b** Axial and **c** coronal CT images show centrilobular, paraseptal, and bullous emphysema predominantly in the upper lobes. **d** Axial image through the lower lobes shows peripheral predominant fibrosis with reticulation, traction bronchiectasis, and honeycombing (black arrow), in a UIP pattern

The histological and radiological patterns of interstitial fibrosis described in CPFE are most commonly UIP and, in a minority of reported cases, NSIP [50]. CPFE is estimated to arise in about 8% of patients diagnosed with IPF [80]. CPFE syndrome is found more frequently in heavy-smoker males and portends a tendency to appear in a slightly older age group in relation to IPF alone (mean age of 65–70 years) ^77-79^. CPFE confers a median survival time nearly double that of IPF (approximately 6.1 years) [81]; however, CPFE portends a higher risk of development of pulmonary hypertension (ranging from 50% to 90%) [82, 83] and may also be associated with a greater chance of lung cancer, with consequent lower survival time [84]. Imaging features are emphysema (both CLE and PSE) in the upper lobes associated with interstitial fibrosis in the lower lobes [31, 50, 76–82, 85, 86]. Ryerson et al. [80] reported that CLE should involve a minimum of 10% of the lung volume to allow the diagnosis. Bulky cystic lesions with thick walls predominantly located in the upper lobes are also apparent, which may correspond to emphysema with fibrosis or AEF (Fig. 20) [85, 86].

## Conclusion

Diffuse smoking-related lung diseases exemplify a wide clinicopathologic manifestation secondary to the same process of lung injury. Histologic findings frequently overlap in a single patient and as a consequence mixed patterns of the disease may be depicted on HRCT. The multidisciplinary approach by an integrated clinical, radiological, and pathological study is useful for the best comprehension and distinction of these entities.

## Data Availability

All data and materials presented were from our hospital and daily practice.

## Abbreviations

AEF: Airspace enlargement with fibrosis
AEP: Acute eosinophilic pneumonia
ALI: Acute lung injury
ARDS: Acute respiratory distress syndrome
BAL: Bronchoalveolar lavage
BMI: Body mass index
CLE: Centrilobular emphysema
COPD: Chronic obstructive pulmonary disease
CPFE: Combined pulmonary fibrosis and emphysema
DIP: Desquamative interstitial pneumonia
DLco: Diffusing capacity for carbon monoxide
GGO: Ground-glass opacity
HRCT: High-resolution computerized tomography
ILD: Interstitial lung disease
IPF: Idiopathic pulmonary fibrosis
LAM: Lymphangioleiomyomatosis
NSIP: Nonspecific interstitial pneumonia
PLCH: Pulmonary Langerhans cell histiocytosis
PLE: Panlobular emphysema
PSE: Paraseptal emphysema
RB: Respiratory bronchiolitis
SRIF: Smoking-related interstitial fibrosis
TWC: Thin-walled cyst
UIP: Usual interstitial pneumonia
